# Supplementary material for: Clinical Instability at Discharge and Post-Discharge Outcomes in Patients with Community-Acquired Pneumonia: An Observational Study
Source: J Clin Med. 2025 Jul 25;14(15):5273. doi: 10.3390/jcm14155273 (PMC12347774; doi:10.3390/jcm14155273)
Supplement: Supplementary file 1 [file jcm-14-05273-s001.zip › jcm-3742392-supplementary.pdf]

Supplementary files

Table S1. Frequency of individual vital sign instability on discharge among CAP patients (n=3984)

**Variable**	**N (%)**
**Temperature ≥37.8 °C**	186 (4.7)
**Heart rate ≥100/min**	427 (10.7)
**Respiratory rate >24/min**	143 (3.6)
**Systolic blood pressure ≤90 mm Hg**	61 (1.5)
**Oxygen saturation of <90%**	175 (4.4)

Table S2. ICD-10-AM codes used to define CAP

ICD-10-AM Code	Name
J13	Pneumonia due to *Streptococcus pneumoniae*
J15	Bacterial pneumonia NEC
J150	Pneumonia due to *Klebsiella pneumoniae*
J151	Pneumonia due to *Pseudomonas*
J152	Pneumonia due to *Staphylococcus*
J153	Pneumonia due to *Streptococcus group B*
J154	Pneumonia due to other *Streptococci*
J155	Pneumonia due to *Escherichia coli*
J156	Pneumonia due to other aerobic gram-negative bacteria
J157	Pneumonia due to *Mycoplasma pneumoniae*
J158	Other bacterial pneumonia
J159	Bacterial pneumonia, unspecified
J16	Pneumonia due to other infectious organisms NEC
J160	Chlamydial pneumonia
J168	Pneumonia due to other specified infectious organisms
J18	Pneumonia, organism unspecified
J180	Bronchopneumonia, unspecified
J181	Lobar pneumonia, unspecified
J182	Hypostatic pneumonia, unspecified
J188	Other pneumonia, organism unspecified
J189	Pneumonia, unspecified

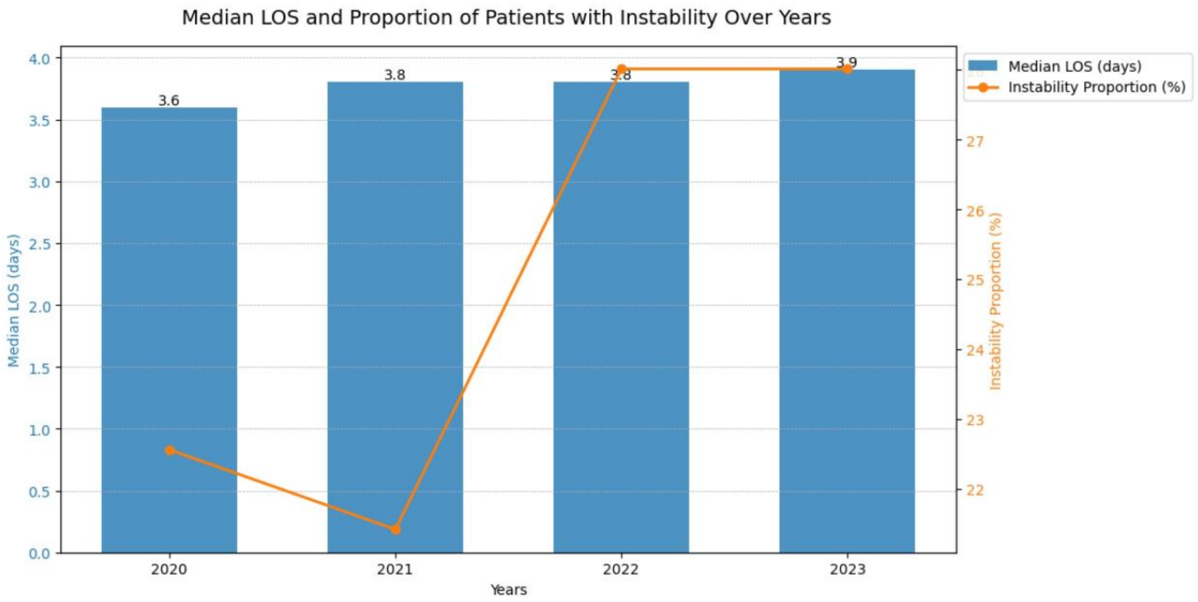

Figure S1. Trend of hospital length of stay and proportion of CAP patients discharged with clinical instability over the four years (p>0.05)

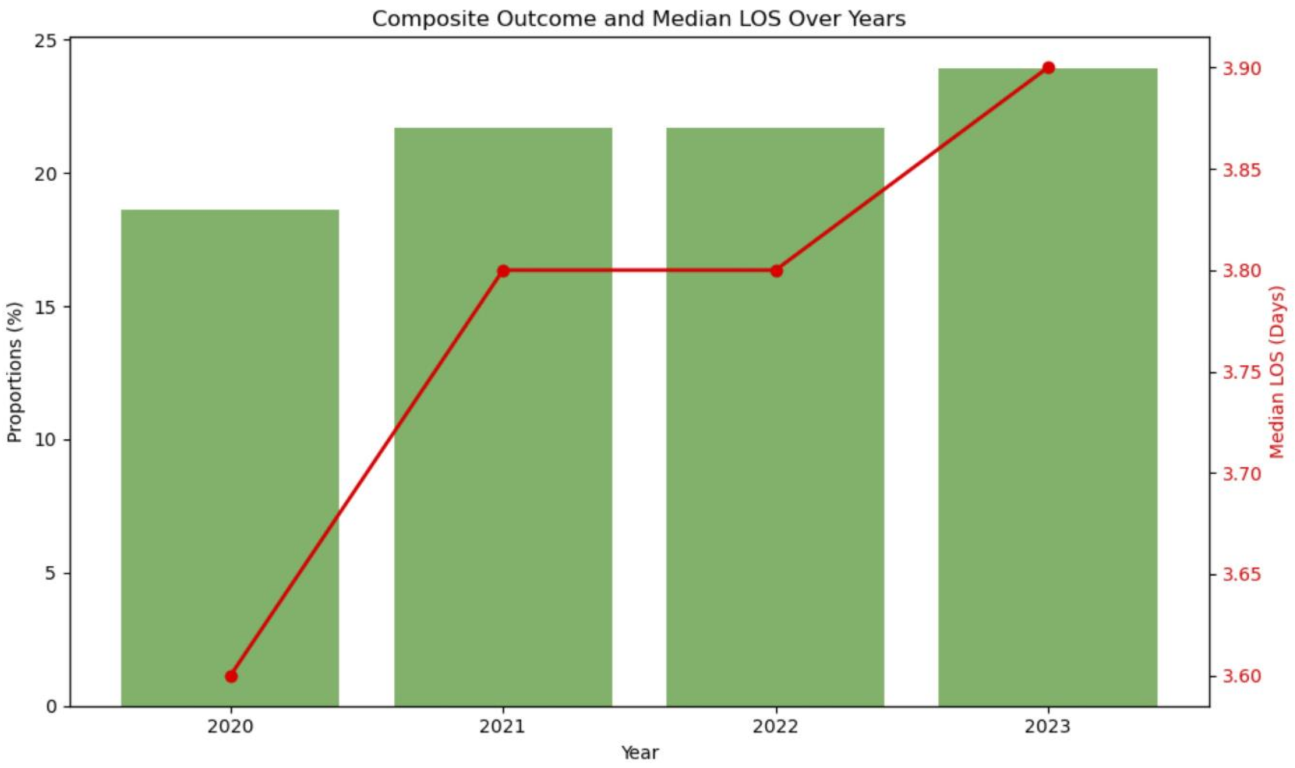

Figure S2. Trends in clinical outcomes and median hospital length of stay among community acquired pneumonia patients discharged over four years (2020–2023) (p>0.05).
